# Strain Variation in Clostridioides difficile Cytotoxicity Associated with Genomic Variation at Both Pathogenic and Nonpathogenic Loci

**DOI:** 10.1128/msphere.00174-22

**Published:** 2022-05-09

**Authors:** Katie Saund, Ali Pirani, D. Borden Lacy, Philip C. Hanna, Evan Snitkin

**Affiliations:** a Department of Microbiology and Immunology, University of Michigan, Ann Arbor, Michigan, USA; b Department of Internal Medicine, Division of Infectious Diseases, University of Michigan, Ann Arbor, Michigan, USA; c Department of Pathology, Microbiology and Immunology, Vanderbilt University School of Medicine, Nashville, Tennessee, USA; d The Veterans Affairs Tennessee Valley Healthcare System, Nashville, Tennessee, USA; University of Iowa

**Keywords:** *Clostridioides difficile*, *Clostridium difficile*, GWAS, bGWAS, WGS, cytotoxins, evolution, genomics, *tcdB*, toxin

## Abstract

Clinical disease from Clostridioides difficile infection can be mediated by two toxins and their neighboring regulatory genes located within the five-gene pathogenicity locus (PaLoc). We provide several lines of evidence that the cytotoxicity of C. difficile may be modulated by genomic variants outside the PaLoc. We used a phylogenetic tree-based approach to demonstrate discordance between cytotoxicity and PaLoc evolutionary history, an elastic net method to show the insufficiency of PaLoc variants alone to model cytotoxicity, and a convergence-based bacterial genome-wide association study (GWAS) to identify correlations between non-PaLoc loci and changes in cytotoxicity. Combined, these data support a model of C. difficile disease wherein cytotoxicity may be strongly affected by many non-PaLoc loci. Additionally, we characterize multiple other *in vitro* phenotypes relevant to human infections, including germination and sporulation. These phenotypes vary greatly in their clonality, variability, convergence, and concordance with genomic variation. Finally, we highlight the intersection of loci identified by the GWAS for different phenotypes and clinical severity. This strategy to identify overlapping loci can facilitate the identification of genetic variation linking phenotypic variation to clinical outcomes.

**IMPORTANCE** Clostridioides difficile has two major disease-mediating toxins, A and B, encoded within the pathogenicity locus (PaLoc). In this study, we demonstrate via multiple approaches that genomic variants outside the PaLoc are associated with changes in cytotoxicity. These genomic variants may provide new avenues of exploration in the hunt for novel disease-modifying interventions. Additionally, we provide insight into the evolution of several additional phenotypes also critical for clinical infection, such as sporulation, germination, and growth rate. These *in vitro* phenotypes display a range of responses to evolutionary pressures and, as such, vary in their appropriateness for certain bacterial genome-wide association study approaches. We used a convergence-based association method to identify the genomic variants most correlated with both changes in these phenotypes and disease severity. These overlapping loci may be important for both bacterial function and human clinical disease.

## INTRODUCTION

Clostridioides difficile is a toxin-producing, healthcare-associated bacterial pathogen. It exhibits extensive genetic variation due to its highly mobile genome, a large pangenome, and a most recent common ancestor for clades C1 to C5 dating back approximately 3.89 million years (1, 2). Such genomic variability has enabled C. difficile adaptation to multiple host species and spread among humans in both nosocomial and community contexts (3). Underlying this genetic variation is phenotypic variation in many traits, including toxin production, sporulation, germination, growth, and virulence (4). This genetic and phenotypic variation has led many to ask whether different genetic backgrounds of C. difficile may differ in their propensity to cause severe infections. To this end, many studies have sought to identify key genetic traits harbored by putative hypervirulent strains, such as ribotype 027 (RT027). Despite this interest and intense study, the genetic basis for variation in phenotypes relevant to the C. difficile infection life cycle remains limited.

Disease during C. difficile infection is mediated by extracellular toxins, primarily toxin A (TcdA) and toxin B (TcdB), which damage the cytoskeletons of intestinal cells, leading to cell death and gut inflammation. These two toxins are large proteins with four domains: glucosyltransferase, autoprotease, pore forming, and C-terminal combined repetitive oligopeptides (CROPs) (5). Toxins A and B are both located within the pathogenicity locus (PaLoc) with three other genes: *tcdR*, *tcdC*, and *tcdE*. *tcdR* is a positive regulator of *tcdA* and *tcdB* and encodes an RNA polymerase factor (6). *tcdC* may be a negative regulator of *tcdR* (6). *tcdE* encodes a holin-like protein and may contribute to toxin secretion (7). Many factors and systems are implicated in PaLoc regulation, including growth phase, access to specific metabolites, sporulation, quorum sensing, and some flagellar proteins (8). In addition to toxin production, other phenotypes may influence C. difficile disease severity or transmission, including sporulation, germination, and growth (9–11).

Approaches for uncovering the genomic determinants of bacterial phenotypes such as cytotoxicity include *in vitro* assays, comparative genomics, and bacterial genome-wide association studies (bGWAS). An advantage of bGWAS is the ability to sift through existing genetic variation in bacterial populations to identify variants associated with natural phenotypic variation. In this way, bGWAS can provide insight into phenotypic evolution and enable the identification of variants of interest that mediate modulation of clinically relevant phenotypes such as virulence (12). Here, we capitalized on a diverse collection of over 100 C. difficile isolates for which multiple phenotypes had previously been characterized (4). We performed whole-genome sequencing and used a bGWAS to uncover novel genotype-phenotype associations. We explore these genotype-phenotype associations and describe the phenotype variation through phylogenetic and evolutionary analyses. Our analyses reveal the influence of genetic variation on phenotypic variation and help illuminate factors that may be influencing clinical disease.

## RESULTS

### Distinct evolutionary trajectories of clinically relevant C. difficile phenotypes.

**FIG 1:**
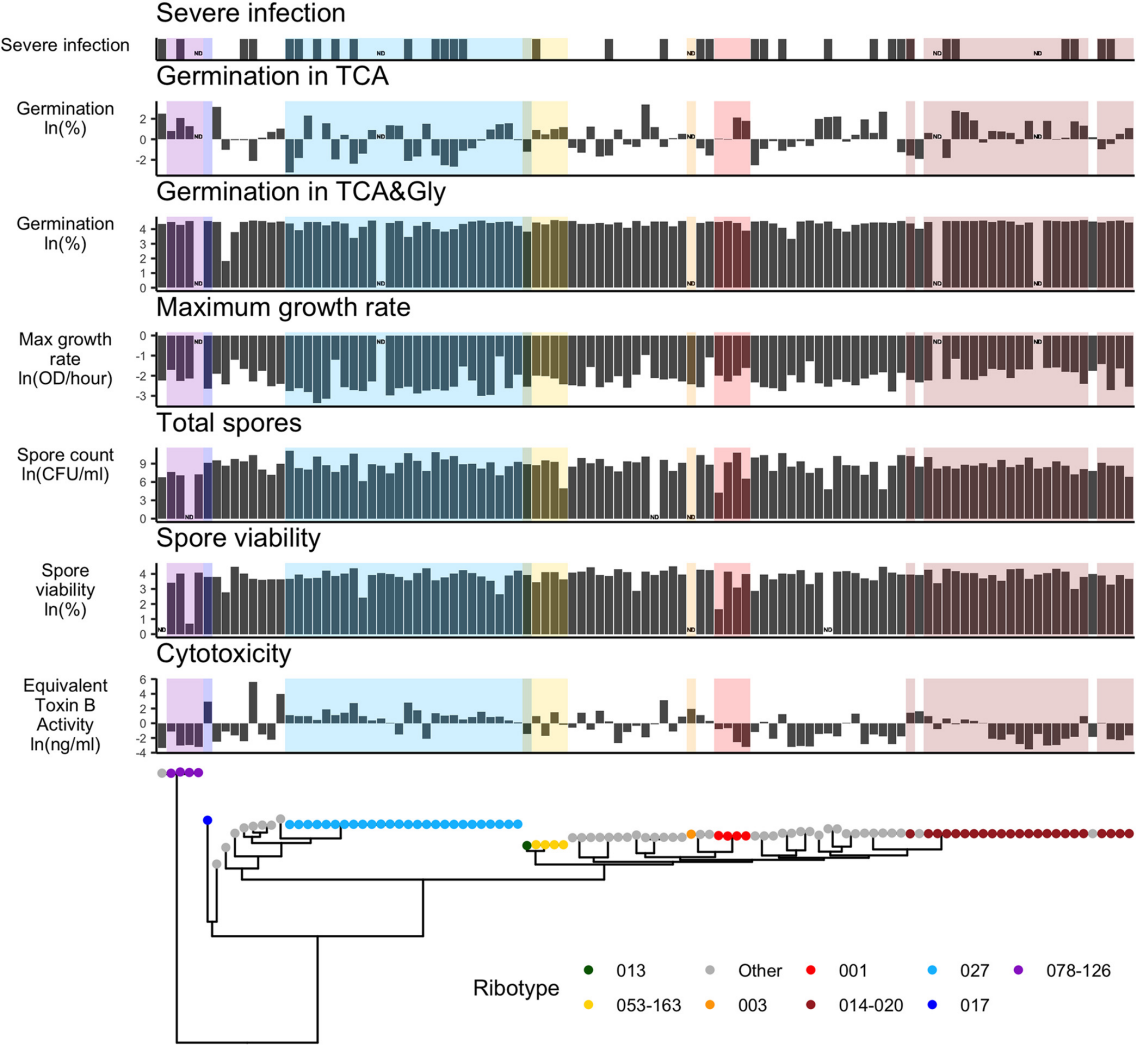
Clinical C. difficile sample phenotypes aligned with the phylogenetic tree. Previous experiments by Carlson et al. characterized germination after a 30-min incubation in 0.1% taurocholate (TCA) (percent), germination after a 30-min incubation in 0.1% TCA and 0.4% glycine (Gly) (percent), maximum growth rates over a 48-h growth period (OD_600_/hour), total spore production (heat-resistant CFU per milliliter), spore viability (percent), and cytotoxicity of the C. difficile supernatant on Vero cells (equivalent toxin B activity in nanograms per milliliter) (4, 10). *In vitro* phenotypes were natural-log transformed. Isolates were collected from stool samples of patients with either severe (bar present) or nonsevere (bar absent) C. difficile infections. Color indicates ribotype. ND, no data.

To improve our understanding of the evolution of phenotypic diversity in C. difficile, we performed whole-genome sequencing on a clinical isolate collection that had previously been assayed for cytotoxicity (a measure that combines the impacts of toxin production, secretion, and activity on Vero cell viability), two measures of germination, two measures of sporulation, and growth rate (4, 10). Overlaying these phenotypes on a whole-genome phylogeny revealed distinct patterns for each phenotype (Fig. 1). Figure 1 displays differences in the variability, degree of convergence, and clonality of these phenotypes. Next, we quantify these differences using a collection of statistical approaches and weave these results into a narrative describing the evolutionary paths for each phenotype.

**FIG 2:**
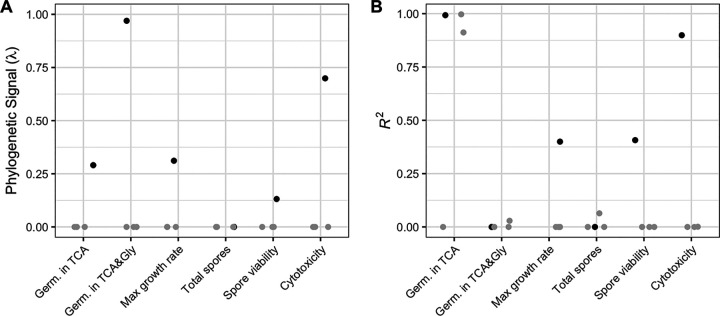
Phenotype phylogenetic signal and genomic model. (A) Phylogenetic signal (λ) of each phenotype (black) or scrambled phenotypes (gray). A near-zero λ value indicates a phenotype that appears independent of the phylogeny. Values of λ near 1 more closely approximate a random walk along the tree. (B) *R*^2^ values from the best linear model per phenotype (black) or scrambled phenotypes (gray). Linear models were generated by an elastic net approach. High *R*^2^ values indicate that the phenotype is strongly encoded by genetic variation in SNPs. Synonymous SNPs were excluded from this analysis.

We quantify phenotype clonality using phylogenetic signal. Phylogenetic signal measures the degree to which closely related samples on a phylogenetic tree are more similar to each other than to random samples. A phenotype whose close neighbors are similar in value but for which random samples are highly variable is said to be modeled well by Brownian motion and has a λ value near 1. In contrast, a phenotype where values are randomly distributed across the tree is modeled well by white noise and has a λ value near zero (13). Cytotoxicity and germination in taurocholate (TCA) and glycine (Gly) are clonal phenotypes that show stable inheritance within lineages, as evidenced by the high phylogenetic signal (Fig. 2A). For example, cytotoxicity displays clonal lineages with uniformly high (e.g., RT027) and low (e.g., RT014) cytotoxicity (Fig. 1). In contrast, germination in TCA and growth rate are less clonal, with extensive variation even within clonal lineages (Fig. 1 and Fig. 2A). Finally, the two sporulation phenotypes show the least clonality, with virtually no clustering on the phylogeny (Fig. 1 and Fig. 2A). Overall, the range in clonality and phylogenetic signal observed for these phenotypes suggests that despite all being central to the C. difficile life cycle, they are shaped by different evolutionary pressures.

In addition to varying in their clonality, the six phenotypes show distinct differences in their overall degree of dispersion (Table 1). Dispersion describes how spread out each phenotype is from its mean. Higher values indicate more variability, and lower values indicate more uniformity. Cytotoxicity had the largest dispersion, with a geometric coefficient of variation of 5.4. The combination of high clonality and high dispersion in cytotoxicity suggests that C. difficile may have evolved multiple successful toxin strategies or may have different evolutionary trajectories that are difficult to escape once begun. In contrast, the near uniformity observed in germination in TCA and Gly could indicate either strong stabilizing selection or inadequate precision of the assay.

**TABLE 1:** Dispersion (geometric coefficient of variation) and convergence (ratio metric of convergence) of the natural-log-transformed phenotypes

	Germination in TCA	Germination in TCA and Gly	Maximum growth rate	Total spores	Spore viability	Cytotoxicity
Geometric coefficient of variation	2.8	0.4	0.5	2.2	0.6	5.4
Ratio metric of convergence	46.8	18.0	43.0	38.7	27.3	33.0

### Phenotypes vary with respect to their association with genetic variation.

Next, we sought to understand the degree to which phenotypic variability in this data set is genetically encoded. We describe this phenotype-genotype relationship with a linear model for each phenotype. In the linear models, a phenotype is the response variable and genomic variants are the explanatory variables. Linear models were constructed via elastic net regularization. We report the *R*^2^ values of the best-fitting model and compare these values to the best values from the negative controls (Fig. 2B). Growth rate, both sporulation phenotypes, and gemination in TCA and Gly have low *R*^2^ values, all <0.50. Germination in TCA has a high *R*^2^ value, 0.99, but this finding appears to be spurious as two of the three negative controls using randomly permuted data have similarly high *R*^2^ values: 0.00, 0.91, and 1.00. The phenotype best modeled by genomic variants is cytotoxicity, with an *R*^2^ value of 0.90 and a distinct separation between the observed value and the values of the negative controls. The germination and total spores phenotypes are so poorly encoded by genomic variation (both *R*^2^ = 0.0) that these assays may lack sufficient precision to capture relevant strain variation, while cytotoxicity appears far more genetically deterministic.

### Phenotypes show a range in their levels of phylogenetic convergence.

A striking feature observed when overlaying the phenotype panel on the whole-genome phylogeny was variation in the frequency of convergence of high or low phenotype values. Convergence, the independent evolution of a trait, may imply the existence of environmental pressures that select for a specific value or constrain the phenotype’s value. To quantify the convergence of the different phenotypes, we employed the ratio metric, where a higher ratio metric value suggests more episodes of convergence. Germination in TCA has the most convergence, at 46.8. The germination in TCA and Gly and spore viability phenotypes have the least convergence, at 18.0 and 27.3, respectively. The remaining phenotypes demonstrate intermediate levels of phylogenetic convergence. There is a striking difference in the convergence of the two germination phenotypes. The low ratio metric in germination in TCA and Gly may be a result of the lack of variability in the phenotype. Germination in TCA and Gly is highly uniform, and thus, there cannot be much convergence to observe. It may be that the permissive laboratory conditions of the germinant TCA plus the cogerminant glycine overpower subtle differences in germination aptitude detectable under the more stringent TCA-only condition. Below, we seek to exploit the high level of convergence in certain phenotypes to identify genetic drivers of their variation.

### Identifying genetic variation associated with phenotypic variation through a genome-wide association study.

Having observed differences in the evolutionary patterns of different phenotypes, we next sought to identify the specific genetic variation that may be underlying phenotypic variation by performing a genome-wide association study (GWAS) for each phenotype. Due to the high convergence in several of the phenotypes (Table 1) and extensive genetic variation in our isolate collection, we opted for a convergence-based GWAS approach that could identify variants of interest by their nonrandom coconvergence with a phenotype. The genotypes tested included approximately 69,600 single nucleotide polymorphisms (SNPs), 8,400 indels, and 7,500 accessory genes. Significantly associated variants were identified for growth rate, total spores, cytotoxicity, germination in TCA, and severity (Table 2).

**TABLE 2:** GWAS results for each phenotype^*a*^

	Germination in TCA	Germination in TCA and Gly	Growth rate	Total spores	Viable spores	Cytotoxicity	Severity
Significant and highly convergent	6	0	10	3	0	8	1,052
Significant	260	0	398	2,894	1,768	220	1,700
Highly convergent	122	0	37	3	2	40	1,117

a Reported values for severity are derived by the Synchronous Test, while all other results are derived by the Continuous Test. Significant loci have an FDR of <15%. Highly convergent loci have an ε value of >0.15.

### Overlapping GWAS results.

**FIG 3:**
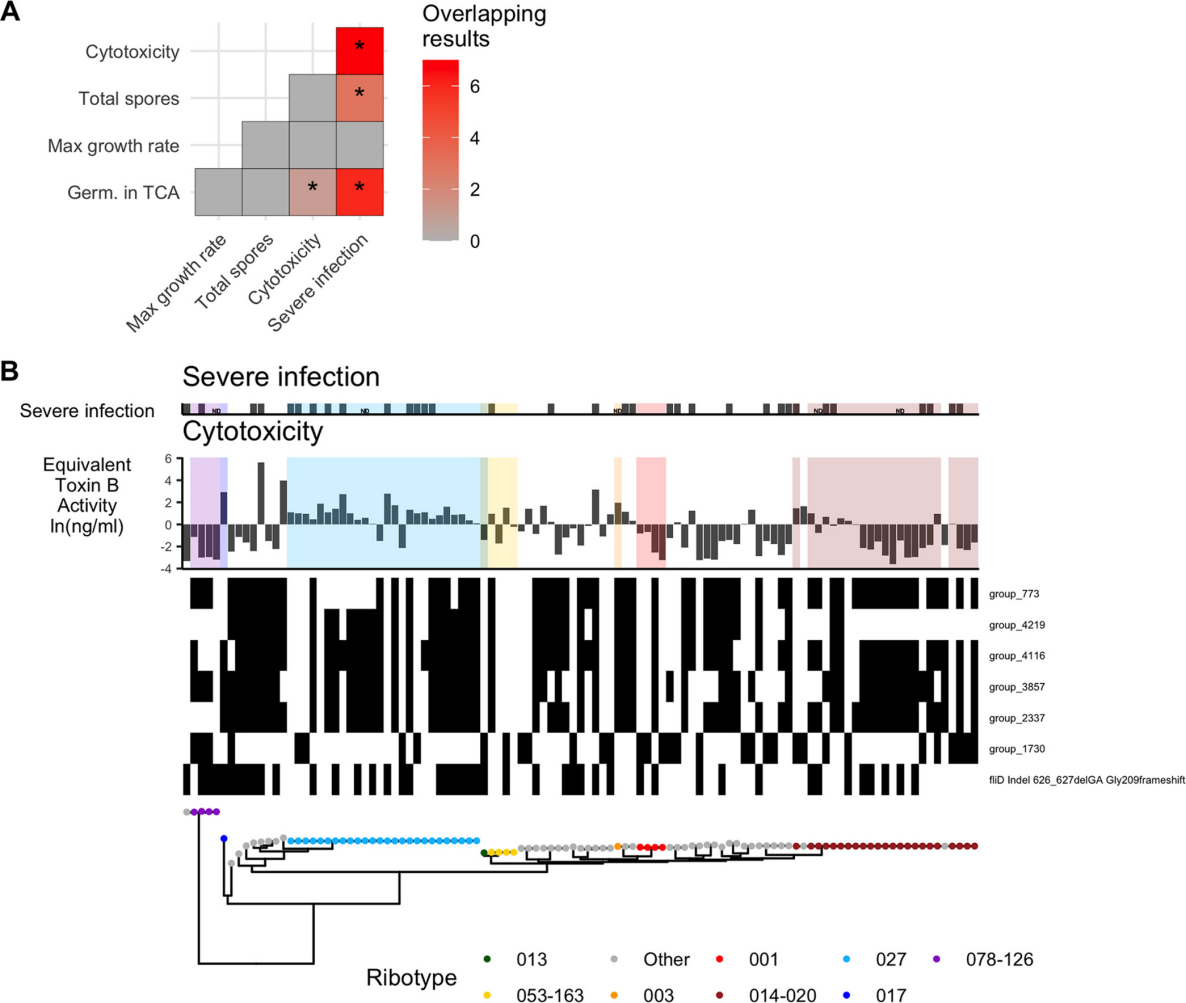
Overlapping GWAS results. (A) Heatmap indicating the number of shared GWAS results with significant *P* values and high levels of convergence in the Continuous Test (continuous phenotypes) or Synchronous Test (severity). Asterisks indicate significantly more overlapping results than expected by chance (*P < *0.05). The two phenotypes lacking any GWAS results with significant *P* values and high levels of convergence were excluded. (B) Shared hits between the cytotoxicity and severe infection GWAS. (Top) Phenotypes. (Middle) Heatmap indicating the presence of loci with both significant *P* values and high levels of convergence in both cytotoxicity and severity GWAS results from panel A. (Bottom) Phylogenetic tree labeled by ribotype.

Despite the phenotypes showing distinct evolutionary patterns, we first explored whether there was evidence of overlap in the genetic circuits modulating the different traits. We cataloged the extent of this overlap by counting the number of intersecting genomic loci with both high significance and convergence in each pair of GWAS results. Three of the four phenotypes shared more hits with the severe-infection GWAS results than expected by chance via a permutation test (Fig. 3A). Cytotoxicity and severe infection have the most overlap, with 7 shared loci. These shared loci include six accessory genes and a frameshift mutation at glycine 209 in flagellar hook-associated protein 2 (*fliD*) (Fig. 3B). The *fliD* finding is consistent with known coregulation that occurs between flagellar and toxin systems in C. difficile that is mediated in part by SigD, a sigma factor that binds to a *tcdR* promoter region and positively regulates *tcdR* (14).

### Genetic variation associated with modulation of cytotoxicity.

For the remainder of our analysis, we focused on understanding genetic variation associated with variation in cytotoxicity. In addition to the central role of toxin in C. difficile disease, our decision to focus on toxin was motivated by it being the phenotype being best explained by genetic variation in sequenced strains (Fig. 2B). In the following sections, we examine variants playing a key role in modulating cytotoxicity.

**FIG 4:**
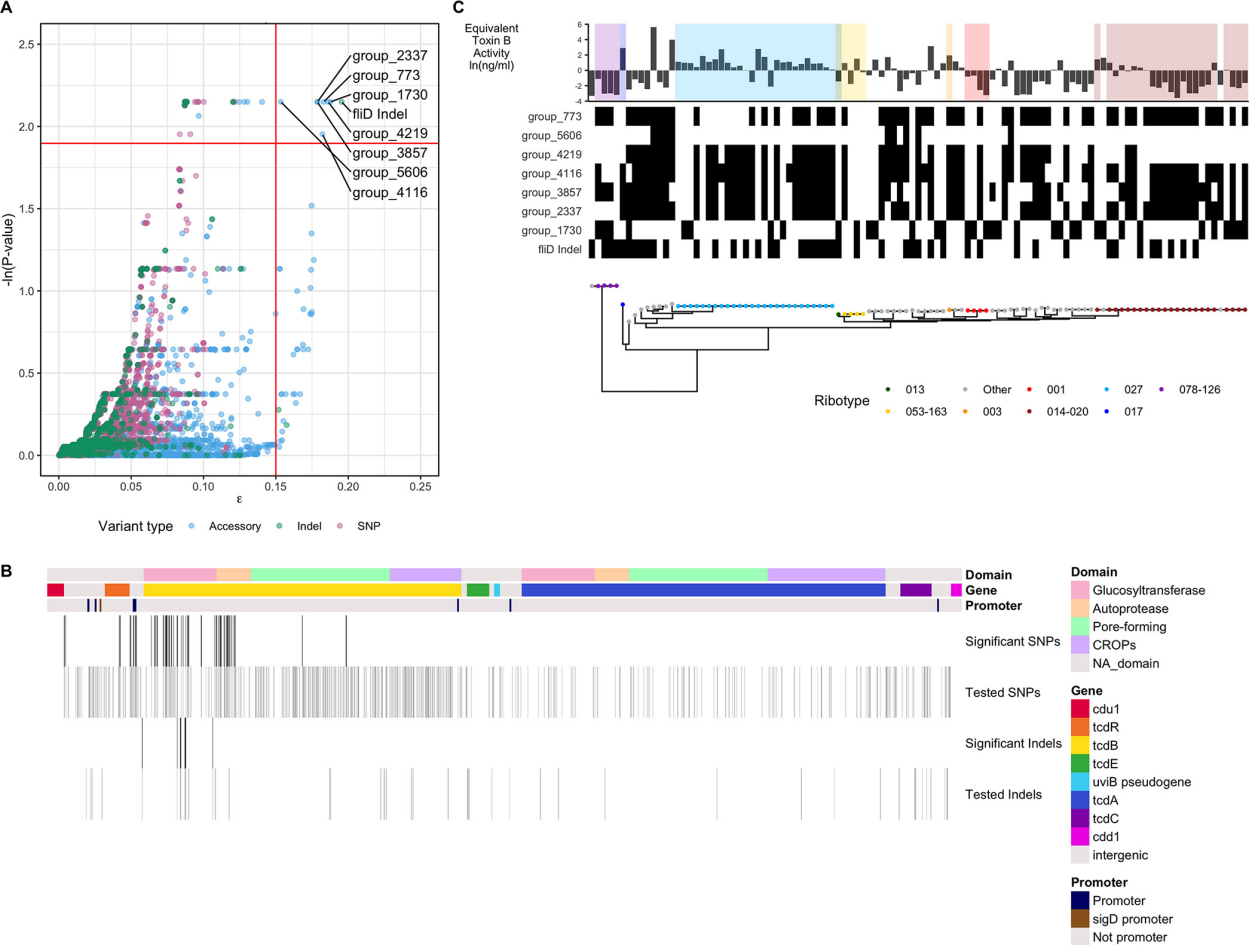
Genome-wide association study identified genomic variants associated with cytotoxicity variation. (A) GWAS results. Tested loci are either accessory genes (blue) (*n* = 4,352), SNPs (pink) (*n* = 12,167), or indels (green) (*n* = 1,843). The red horizontal line indicates a false discovery rate of 15%. The red vertical line separates low from high convergence. (B) Significantly associated loci from the GWAS located in the PaLoc. Of the 633 PaLoc variants (SNPs, *n* = 563; indels, *n* = 70) tested by the GWAS, only the variants significantly associated are plotted as vertical bars (SNPs, *n *= 71; indels, *n* = 16). (Top annotation) Toxin protein domains in *tcdA* and *tcdB*. (Middle annotation) Gene. (Bottom annotation) Promoter locations. (C, left) Phylogenetic tree labeled by ribotype. (Middle) Heatmap indicating the presence of loci significantly associated with cytotoxicity and with high convergence. (Right) Cytotoxicity.

The cytotoxicity GWAS identified many genomic variants of interest. Two hundred twenty loci were significantly associated with cytotoxicity changes (above the horizontal red line), 40 loci had high levels of convergence (right of the vertical red line), and 8 loci both were significantly associated and had high levels of convergence (upper right quadrant) (Fig. 4A). As the PaLoc harbors toxin genes and regulators, we expected that variants located within the PaLoc would be significantly associated with cytotoxicity and used this as a positive control for our analysis. Consistent with this, we observed PaLoc variants in the pool of significant results associated with cytotoxicity. Eighty-seven of the 220 loci significantly associated with cytotoxicity occur in the PaLoc. Given that the cytotoxicity assay used is based on a standard curve measuring toxin B activity, it is particularly striking that these 87 PaLoc loci include 75 *tcdB* variants and 2 *tcdR*-*tcdB* intergenic region variants but no variants within *tcdA* (Fig. 4B). Indeed, these variants are significantly enriched compared to the number of variants within or flanking *tcdB* that are expected by chance using a permutation approach (*P* = 0.0001) (median = 1; range = 0 to 10). *tcdB* variants were found in all four protein domains, but the significantly associated variants are found mostly within the glucosyltransferase and autoprotease domains (Fig. 4B). Certain significant missense variants within *tcdB* have plausible functional impacts on toxin B, such as an adenosine-to-cytosine transversion at position 1967 that changes an aspartic acid to alanine (*P* = 0.12); this mutation occurs near the zinc binding site and could theoretically affect toxin autoprocessing within the host cell. Of the 15 tested variants that occur within the *tcdR*-*tcdB* intergenic region, 6 were significantly associated with cytotoxicity (all *P* ≤ 0.12). Three of these variants occur within a *tcdB* promoter, suggesting a potential role in modulating sigma factor binding and therefore altering *tcdB* transcription. A notable lack of association was observed for an adenosine deletion at nucleotide 117 in *tcdC* that has been suggested to cause increased toxin production in RT027 (*P* = 0.95) (15). This deletion was found in all 26 RT027 samples as well as 3 additional samples (“other” ribotype) but did not reach significance in the GWAS (*P* = 0.95).

Next, we sought to generate hypotheses about new associations between genomic variants and cytotoxicity that reside outside the PaLoc. The 8 variants that were significant and had a high ε value, a metric of shared genotype-phenotype convergence, are cataloged in Data Set S1 in the supplemental material, plotted in Fig. 4C, and listed in Table 3 (16). A single ε value captures the number of tree edges where both a genotype is mutated and the cytotoxicity value has a large change. ε values close to zero suggest that the genotype mutates on very few edges where the cytotoxicity changes drastically. The loci associated with changes in cytotoxicity are present in multiple, independent lineages (Fig. 4C). The above-mentioned frameshift mutation in *fliD* is the variant most strongly associated with changes in cytotoxicity when ranked by ε and then *P* value (*P *= 0.12; ε = 0.20). The next most strongly associated variant is an accessory gene, “group_1730,” generated by the pangenome detection tool roary (*P *= 0.12; ε = 0.19). This accessory gene is orthologous to CD630_21340, which is annotated as a diguanylate cyclase/phosphodiesterase in the CD630 reference genome. The other most strongly associated variants are unannotated accessory genes and CD630_18290 (*kdpD*), a histidine kinase. The significant accessory genes identified by this analysis represent candidates for future mechanistic studies dissecting C. difficile cytotoxicity and could be prioritized for further characterization.

**TABLE 3:** Cytotoxicity GWAS results^*a*^

Locus	*P* value	ε	Annotation	Variant type	Rank
*fliD* (deletion at positions 626–627 leading to frameshift mutation)	0.11	0.20	Flagellar hook-associated protein 2	Deletion	1
Group_1730	0.11	0.19	GGDEF domain	Accessory gene	2
Group_4219	0.11	0.19	None assigned	Accessory gene	3
Group_773	0.11	0.18	None assigned	Accessory gene	4
Group_4116	0.14	0.18	None assigned	Accessory gene	5
Group_2337	0.11	0.18	None assigned	Accessory gene	6
Group_3857	0.11	0.18	CD630_18290; *kdpD*; ATPase histidine kinase DNA gyrase B HSP90 domain	Accessory gene	7
Group_5606	0.11	0.15	None assigned	Accessory gene	35

a Loci have an FDR of <15% and an ε value of >0.15. Locus names for accessory genes were generated by roary. Where possible, additional gene annotations are provided.

10.1128/msphere.00174-22.1 DATA SET S1 Cytotoxicity GWAS results. Variant names, *P* values, and ε values are included (continued in Data Set S2 in the supplemental material). Download Data Set S1, TXT file, 2.5 MB. Copyright © 2022 Saund et al. 2022 Saund et al. https://creativecommons.org/licenses/by/4.0/ This content is distributed under the terms of the Creative Commons Attribution 4.0 International license.

### Genetic variation at the PaLoc accounts for only half of the phenotypic variation in cytotoxicity.

**FIG 5:**
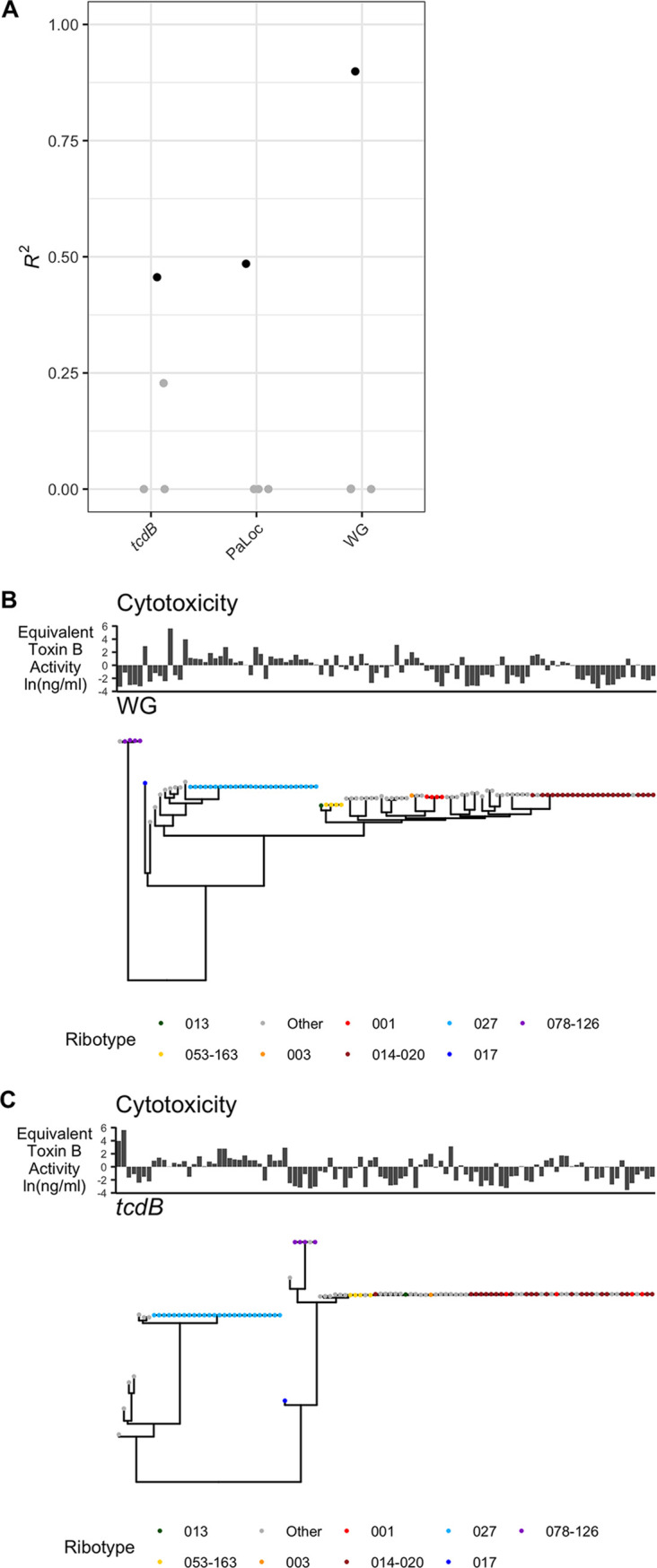
*tcdB* variation does not fully model cytotoxicity. (A) Elastic net model performance of cytotoxicity. Models were built from *tcdB* variants, PaLoc variants, or whole-genome (WG) variants. (B and C) Cytotoxicity with a tree built from the whole genome (B) or just *tcdB* (C).

The GWAS identified both PaLoc and non-PaLoc loci correlated with variation in cytotoxicity. To understand the relative contribution of genetic variation in the PaLoc to variation in cytotoxicity, we employed an elastic net approach. Models of cytotoxicity constructed with different subsets of variants found that PaLoc variants and *tcdB* variants have similar abilities to model cytotoxicity (*R*^2^ = 0.48 and *R*^2^ = 0.46, respectively) (Fig. 5A). However, variants from the whole genome build a more accurate model of cytotoxicity (*R*^2^ = 0.90) (Fig. 5A). Of the 634 variants in the PaLoc, 404 (64%) occur in *tcdB* or its flanking intergenic regions; in the best-performing elastic net model derived from PaLoc variants, 34/61 (56%) of the variants are mutations in *tcdB* or its flanking regions. In the whole-genome model, only 17/1,795 (1%) of the variants occur in *tcdB* or its flanking regions.

To assess the capacity of PaLoc variation to model cytotoxicity in a different way, we compared phylogenetic trees built from whole-genome variants and the *tcdB* gene. As there is far less variation in *tcdB* than in the whole genome, we observe many polytomies in the *tcdB* gene tree and none in the whole-genome tree (Fig. 5B and C). While the *tcdB* gene tree’s cytotoxicity is better modeled by Brownian motion (λ = 0.94) than in the whole-genome tree (λ = 0.75) (Fig. 2A), there remains much cytotoxicity variation unexplained by tree structure. Given the unexplained cytotoxicity variation on the *tcdB* gene tree and variation not captured in the cytotoxicity elastic net model, we conclude that while *tcdB* gene variation is likely an important mediator of the evolution of cytotoxicity, other loci play a key role as well. Finally, the whole-genome model suggests that many loci besides *tcdB* may affect C. difficile toxicity. This finding is consistent with reports that some non-PaLoc loci contribute to toxin regulation (8, 17–19).

## DISCUSSION

C. difficile is a genetically diverse pathogen, with extensive variation in both its core and accessory genome. Currently, we have a limited understanding of the functional impact of most of this variation and how it relates to C. difficile infection. Here, we attempted to improve our understanding of the genotype-to-phenotype map in C. difficile by analyzing variation in clinically relevant phenotypes in the context of C. difficile genomic variants. We observe that despite their central role in the C. difficile transmission-and-infection cycle, sporulation, germination, growth, and cytotoxicity show distinct evolutionary trajectories. Focusing on the phenotype thought to be most closely linked to virulence, we observe that cytotoxicity is highly clonal, with lineages tending to possess either high or low cytotoxicity. Consistent with previous reports, we find that variation in toxicity can be modulated by variants in the PaLoc; however, we find that more than 50% of the phenotypic variation is associated with genetic variation outside the PaLoc.

Our exploration of these C. difficile phenotypes revealed a broad range of clonality, dispersion, association with genomic variation, and convergence. As such, each phenotype appears to be shaped by different selection forces. The existence of phenotypes that show no association with the recombination-filtered phylogeny could indicate either a lack of precision in the laboratory assay or a strong role for recombinant genomic regions in shaping these phenotypes. We focused our analysis on cytotoxicity, in part, because of the precision of the *in vitro* assay results and its high degree of genetic determinism. Regardless of the basis for the lack of phylogenetic signal in some of the nontoxin phenotypes, these results show how overlaying phenotypic variation on whole-genome phylogenies provides useful context for interpreting and scrutinizing experimental measurements and, in this case, clearly demonstrates the rich and varied patterns of evolution among C. difficile strains.

Toxigenic bacterial species that require live transmission may undergo strong selective pressure to promote host survival and therefore bias toward lower toxin activity (20). In contrast, sporogenic C. difficile can survive and transmit even after the host dies; this may reduce the strength of selection on toxicity, and therefore, many different toxin strategies are successful. Indeed, there are prolific toxigenic and nontoxigenic strains of C. difficile. Additionally, the species has had multiple independent losses of the PaLoc (21), with our results indicating that even strains harboring an intact PaLoc may evolve to have decreased cytotoxicity. The C. difficile strains with high cytotoxicity may have success by shaping a hostile metabolic state in the host gut that these bacteria are able to uniquely exploit (22) or its more severe, inflammatory infection, which results in diarrhea and, therefore, increased transmission. This then raises the question of what the selective pressure for lower cytotoxicity may be. One possibility is that cytotoxicity itself may not be the most critical aspect of the toxin upon which evolution is acting, with other aspects such as toxin immunogenicity potentially evoking a stronger selection pressure. Toxin that evades immune recognition could lead to longer infections and, therefore, increased transmission, so the strongest selective pressure may be at the surface domains of the toxin proteins rather than on regulators of toxin activity (21). For example, we observed multiple missense variants on the surface of *tcdB* in this isolate collection, including a glutamic acid 329-to-glycine missense variant and a threonine 430-to-alanine variant.

Our study has several important limitations. First, the limited sample size of this C. difficile collection could lead to an underreporting of the clonality of some phenotypes for underrepresented ribotypes and limit the power to detect variation with smaller phenotypic impacts. Second, many genomic features such as copy number variants, large structural variants, and plasmids were not included in our GWAS or elastic net models; therefore, these analyses are missing some genome-encoded information. Similarly, we were not able to capture the impact of genetic switches, such as the small inversion that acts as a dynamic flagellar switch, which has been shown to impact toxin gene expression and toxin secretion (19). We also did not consider the impact of epistatic interactions between genomic variants on phenotypes. Third, the toxin assay used measures the impact of the C. difficile supernatant on Vero cells. Therefore, this assay measures the combined impacts of toxin production, toxins secreted into the supernatant, and the activity of the toxin proteins. Future work could separate the impacts of genomic variants on toxin secretion and toxin activity.

A replication study in a second C. difficile cohort in which the toxin assay and GWAS are repeated could help prioritize the genomic variants more likely to be causal of changes in cytotoxicity. The loci identified in both this study and the proposed study would be higher-confidence candidates for experiments that examine the effect of those potential variants on cytotoxicity. Additional studies investigating C. difficile *in vitro* phenotypes from an evolutionary perspective would help to prioritize the phenotypes that may offer the most insight into the success and regulation of certain strains.

## MATERIALS AND METHODS

### Study population.

The University of Michigan Institutional Review Board approved all sample and clinical data collection protocols used in this study (HUM00034766). Where applicable, written, informed consent was received from all patients prior to inclusion in this study. Stool samples were collected from a cohort of 106 Michigan Medicine patients with C. difficile infection from 2010 to 2011, which included all severe cases during the study period (4, 10). Cases were classified as severe if the infection required intensive care unit (ICU) admission or interventional surgery or if the patient died within 30 days of infection diagnosis. A clonal spore stock from each patient was used for ribotyping and *in vitro* studies.

### *In vitro* characterization.

Previous experiments characterized the *in vitro* qualities of the isolate collection (4, 10). Below, we briefly summarize each assay (for further details, see references 4 and 10). Taurocholate (TCA) is a physiologic bile salt known to cause C. difficile germination; glycine is a cogerminant that can increase germination with taurocholate (23). Germination was performed in 0.1% TCA for 30 min. After a 1:10 dilution to halt further germination, samples were serially diluted and plated onto brain heart infusion-supplemented (BHIS) with or without 0.1% TCA. CFU counts are reported as the percentage of BHIS only/BHIS plus 0.1% TCA. The germination assay was repeated in 0.1% TCA and 0.4% glycine. Maximum growth rates (optical density at 600 nm [OD_600_]/hour) were calculated from OD readings taken every 10 min over a 48-h period. Total spore production, defined as heat-resistant CFU per milliliter, was calculated as CFU per milliliter after (i) a 24-h growth period followed by (ii) a heat treatment (65°C for 30 min) to kill vegetative cells and finally (iii) plating. Spore viability is reported as the percentage of CFU/spores plated. The cytotoxicity assay measured the effect of the cell-free, toxin-containing C. difficile supernatant on Vero cell viability. It measured the combined impacts of toxin production, secretion, and activity. A standard curve was produced by exposing Vero cells to known quantities of toxin B (nanograms per milliliter). Samples were classified as severe infections if they were collected from a patient whose C. difficile infection required ICU admission or interventional surgery or if the patient died within 30 days of infection diagnosis (4, 10).

### Genomic analysis.

The spore stocks were grown in an anaerobic chamber overnight on taurocholate-cefoxitin-cycloserine-fructose agar plates. The next day, a single colony of each sample was picked and grown in brain heart infusion medium with yeast extract liquid culture medium overnight. The vegetative C. difficile cells were pelleted by centrifugation and washed, and total genomic DNA was then extracted. Genomic DNA extracted with the MoBio PowerMag microbial DNA isolation kit (Qiagen) from C. difficile isolates (*n* = 108) was prepared for sequencing using the Illumina Nextera DNA Flex library preparation kit. Sequencing was performed on either an Illumina HiSeq 4000 system at the University of Michigan Advanced Genomics Core or an Illumina MiSeq system at the University of Michigan Microbial Systems Molecular Biology Laboratories. The quality of reads was assessed with FastQC v0.11.9 (24). Adapter sequences and low-quality bases were removed with Trimmomatic v0.36 (25). Variants were identified by mapping filtered reads to the CD630 reference genome (GenBank accession number AM180355.1) using bwa v0.7.17 (26), removing PCR duplicates with Picard 2.21.7 (27), removing clipped alignments using Samclip 0.4.0 (28), and calling variants with SAMtools v1.11 and bcftools (29). Variants were filtered from raw results using GATK’s VariantFiltration v3.8 (quality score [QUAL], >100; root mean square mapping quality [MQ], >50; ≥10 reads supporting the variant; consensus quality [FQ] <0.025) (30). SNPs and indels were referenced to the ancestral allele using snitkitr v0.0.0.9000 (31). Pangenome analysis was performed with roary (32). Annotations were assigned by prokka v1.14.5 (33) and emapper-2.1.7 based on eggNOG orthology data (34, 35). Sequence searches were performed using DIAMOND (36). Gene prediction was performed using Prodigal (37). Gene annotation results are accessible in Data Set S7 in the supplemental material. Binary toxin genes, *cdtA* and *cdtB*, were not identified in the pangenome generated from this collection, and therefore, binary toxin was excluded from the analysis.

10.1128/msphere.00174-22.7 DATA SET S7 Key mapping pangenome to gene annotations. Download Data Set S7, TXT file, 0.8 MB. Copyright © 2022 Saund et al. 2022 Saund et al. https://creativecommons.org/licenses/by/4.0/ This content is distributed under the terms of the Creative Commons Attribution 4.0 International license.

### Phylogenetic analysis.

Consensus files generated during variant calling were recombination filtered using Gubbins v3.0.0 (38). The alleles at each position that passed filtering were concatenated to generate a noncore variant alignment relative to the CD630 reference genome. Alleles that did not pass filtering were considered unknown (denoted N in the alignment). Variant positions in the alignment were used to reconstruct a maximum likelihood phylogeny with IQ-TREE v1.5.5 using ultrafast bootstrapping with 1,000 replicates (39, 40). ModelFinder limited to ascertainment bias-corrected models was used to identify the best nucleotide substitution model (41). The tree was midpoint rooted. The *tcdB* multiple-sequence alignment was built by PRANK v.170427 using only the *tcdB* gene, and the resulting tree was midpoint rooted (42). The trees are available in Data Sets S5 and S6.

10.1128/msphere.00174-22.5 DATA SET S5 Phylogenetic tree. Download Data Set S5, TXT file, 0.01 MB. Copyright © 2022 Saund et al. 2022 Saund et al. https://creativecommons.org/licenses/by/4.0/ This content is distributed under the terms of the Creative Commons Attribution 4.0 International license.

10.1128/msphere.00174-22.6 DATA SET S6 *tcdB* gene phylogenetic tree. Download Data Set S6, TXT file, 0.01 MB. Copyright © 2022 Saund et al. 2022 Saund et al. https://creativecommons.org/licenses/by/4.0/ This content is distributed under the terms of the Creative Commons Attribution 4.0 International license.

### Genome-wide association studies.

GWAS were performed with hogwash v1.2.4 (16). Phenotype data were natural-log transformed. Hogwash settings were a bootstrap threshold of 0.95, 10,000 permutations, and a false discovery rate (FDR) of 15%. The analysis included SNPs, indels, and accessory genes. The intersection of hogwash results was restricted to results with an ε value of >0.15 and a *P* value of <0.15. Only SNPs classified as having a “moderate,” “high,” or “modifier” impact by SnpEff v4.3.1 were included (43).

### Data analysis.

Data analysis with R v3.6.2 (44) was performed with the following packages: ape v5.3 (45), aplot v0.0.6 (46), data.table v1.12.8 (47), ggtree v2.0.4 (48), ggpubr v0.4.0 (49), pheatmap v1.0.12 (50), phytools v0.6-99 (51), and tidyverse v1.3.0 (52). Conda v4.9.2 was used to maintain working environments (53). Analysis code is available at https://github.com/katiesaund/cdifficile_gwas.

### Permutation testing.

The empirical *P* values for enrichment of toxin variants in the significant GWAS results and shared results in the overlapping GWAS section were generated via permutation testing. This approach generates a *P* value by comparing the observed number of events in the data to a distribution of the number of events simulated under the null hypothesis. The null distribution was generated from random sampling without replacement repeated in 10,000 trials (toxin variants) or 1,000 trials (overlapping hits). Multiple-testing correction was applied to the overlapping-hit analysis using Bonferroni correction.

### Convergence analysis.

We calculated the degree of convergence of each phenotype using the ratio method (54), which is the ratio of two samples’ pairwise patristic distance divided by their pairwise phenotypic distance. We report the average of the scaled pairwise branch length distance (patristic distance) divided by the scaled pairwise phenotypic distance for each phenotype. A high value suggests an episode of convergence.

### Geometric coefficient of variance.

To capture the dispersion of the data sets, we calculated the geometric coefficient of variance, $\sqrt{e^{{\text{σ}}^{2}}-1}$, where σ is the standard deviation of the natural-log-transformed data. This metric quantifies the degree to which each phenotype is spread out from its mean.

### Phylogenetic signal.

Phylogenetic signal is a metric that captures the tendency for closely related samples on a tree to be more similar to each other than they are to random samples on the tree. We calculated phylogenetic signal for each continuous phenotype using Pagel’s λ (13). Note that a phenotype that is modeled well by Brownian motion has a λ value near 1. Such a phenotype is strongly reflected in the tree structure. A phenotype modeled well by white noise has a λ value near zero (13). Such a phenotype is randomly distributed on a tree and therefore appears to be independent of the phylogeny. Negative controls for the phenotypes were created by randomly redistributing each phenotype on the tree.

### Elastic net modeling.

We calculated the degree of genetic encoding of each phenotype by modeling a phenotype from genomic variants using elastic net regularization as implemented by pyseer. pyseer v1.3 command line arguments were as follows: –wg enet –n-fold 10 (55). The model was trained using 10-fold cross-validation. We did not use the built models for prediction. We report the best *R*^2^ values from cross-validation. SNPs, indels, and accessory genes were all used to model all continuous phenotypes. For all elastic net models, only SNPs classified as having a “moderate,” “high,” or “modifier” impact by SnpEff were included (43). Cytotoxicity was additionally modeled by (i) a model built from just PaLoc SNPs and indels and (ii) a model built from just *tcdB* SNPs and indels. To determine the value of α, a parameter that controls the ratio of L1 and L2 regularization in the model, five α values were tested for each model: 0.01, 0.245, 0.500, 0.745, and 0.990. The model results with the highest *R*^2^ values are reported. The best α value for models of germination in TCA, germination in TCA and Gly, total spores, and cytotoxicity (all variants) is 0.01. The best α value for models of viable spores and growth rate is 0.245. The best α value to model cytotoxicity (*tcdB*) is 0.500. The best α value to model cytotoxicity (PaLoc) is 0.745. Negative controls for the phenotypes were created by randomly redistributing each phenotype on the tree.

### Data availability.

Sequence data are available under BioProject accession number PRJNA594943. Details on sequenced strains are available in Data Set S3 in the supplemental material. Sequences for genes identified by roary are available in Data Set S4.

10.1128/msphere.00174-22.2 DATA SET S2 Remaining cytotoxicity GWAS results. Variant names, *P* values, and ε values are included. Download Data Set S2, TXT file, 3.1 MB. Copyright © 2022 Saund et al. 2022 Saund et al. https://creativecommons.org/licenses/by/4.0/ This content is distributed under the terms of the Creative Commons Attribution 4.0 International license.

10.1128/msphere.00174-22.3 DATA SET S3 BioProject details for the sequenced strains. Download Data Set S3, TXT file, 0.02 MB. Copyright © 2022 Saund et al. 2022 Saund et al. https://creativecommons.org/licenses/by/4.0/ This content is distributed under the terms of the Creative Commons Attribution 4.0 International license.

10.1128/msphere.00174-22.4 DATA SET S4 Sequences of the genes identified by roary. Download Data Set S4, TXT file, 7.8 MB. Copyright © 2022 Saund et al. 2022 Saund et al. https://creativecommons.org/licenses/by/4.0/ This content is distributed under the terms of the Creative Commons Attribution 4.0 International license.
